# Environmental Physiology and Diving Medicine

**DOI:** 10.3389/fpsyg.2018.00072

**Published:** 2018-02-02

**Authors:** Gerardo Bosco, Alex Rizzato, Richard E. Moon, Enrico M. Camporesi

**Affiliations:** ^1^Environmental Physiology and Medicine Lab, Department of Biomedical Sciences, University of Padova, Padua, Italy; ^2^Center for Hyperbaric Medicine and Environmental Physiology, Department of Anesthesiology, Duke University Medical Center, Durham, NC, United States; ^3^TEAMHealth Research Institute, Tampa General Hospital, Tampa, FL, United States

**Keywords:** diving, oxygen, decompression sickness, pulmonary edema, gas exchange

## Abstract

Man’s experience and exploration of the underwater environment has been recorded from ancient times and today encompasses large sections of the population for sport enjoyment, recreational and commercial purpose, as well as military strategic goals. Knowledge, respect and maintenance of the underwater world is an essential development for our future and the knowledge acquired over the last few dozen years will change rapidly in the near future with plans to establish secure habitats with specific long-term goals of exploration, maintenance and survival. This summary will illustrate briefly the physiological changes induced by immersion, swimming, breath-hold diving and exploring while using special equipment in the water. Cardiac, circulatory and pulmonary vascular adaptation and the pathophysiology of novel syndromes have been demonstrated, which will allow selection of individual characteristics in order to succeed in various environments. Training and treatment for these new microenvironments will be suggested with description of successful pioneers in this field. This is a summary of the physiology and the present status of pathology and therapy for the field.

## Introduction

Exploration of underwater environments predates documentation for possibly 1000s of years, but written evidence dates back to Ancient Greece in the 4th century BCE; Aristotle recounts the use of a primitive diving bell, an overturned cauldron that trapped air from the atmosphere. Today diving is incorporated into many aspects of modern society including: sports, recreation, industry, and widespread strategic military goals. Subaquatic environments are an essential piece of modern day life, and will only play a bigger role in the future. Within the last century, there has been growing interest in diving for both recreational and professional purposes. The worldwide number of annual diving certifications has tripled during the past 20 years, and in the United States alone there are an estimated 4 million sport divers (Lynch and Bove, 2009). Diving will change rapidly again in the coming years, with plans to establish permanent underwater installations that have specific research goals of exploration, health, and survival.

Profound pulmonary, circulatory, and cardiac changes are induced by immersion in water; divers must tolerate and compensate for changes in pressure and temperature. In this review, we will discuss suggested treatments and preventive training for these new microenvironments as gleaned from successful pioneers; we will summarize the physiology and the present status of pathology and therapy in the field; and we will illustrate the physiological changes induced by immersion, swimming, breath-hold diving, and exploring while using special equipment in the water.

Only in the last 100 years has technology allowed people to reach extreme depths using compressed gas delivery devices and diving suits containing air at atmospheric pressure. Free-diving has also been achieved at considerable depths (Balestra and Germonpré, 2014). We will review the major environmental challenges at these depths: density, pressure, temperature. Then we will briefly explain the physiological changes induced by immersion and discuss the clinical implications of diving. When underwater the human body is challenged by forces and physiological concerns not faced in its every-day environment. Familiarity with the risks involved with diving and mindfulness are critical to maintain one’s health during diving exposure (Lanphier, 1957).

## The Water Environment

Understanding the physical properties of the underwater environment remains the best approach to minimize risks while diving when breathing support systems are needed (Pendergast and Lundgren, 2009; Pendergast et al., 2015). The hazardous characteristics of submersion are: density, pressure, temperature, and optical phenomena.

### Density

Flotation in any fluid is ruled by the density of water, 1 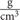, (Baronti et al., 2012; Pendergast et al., 2015). According to Archimedes’ law objects denser than water will sink. As seawater has a high salinity the density is closer to 1.029 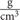 at the surface. Hence, it is easier to float in saltwater as it is denser than fresh water. Net buoyancy (equal to the magnitude of the weight of fluid displaced by the body) of breath-hold divers (humans or animals) affects their swimming behavior and energetics (Williams et al., 2000). However, the high density of water implies an enhanced energy cost of locomotion, reducing the human efficiency in the water environment (Bosco et al., 2014; Pendergast et al., 2015). A diver’s overall density depends upon incompressible non-gas tissues (tissue density), and the volume and density of highly compressible gas stores carried within the body (i.e., natural cavities such as lungs, stomach and other viscera) (Baronti et al., 2012). Another important parameter, strictly related to density is dynamic viscosity. It determines how easily bodies move through water and divided by density represents kinematic viscosity (Pendergast et al., 2015).

Moreover, density of the inspired gas determines breathing resistance. During immersion using underwater breathing apparatus, air (or any other respiratory mixture) is delivered at the diver’s ambient pressure. Breathing gas density increases in direct proportion to ambient pressure, with a corresponding increase in work of breathing. For this reason, helium-oxygen composite mixtures used during deeper dives to eliminate the narcotic effects of nitrogen also reduce breathing resistance (Brubakk et al., 2003; Balestra and Germonpré, 2014).

### Pressure

Small pressure effects on biological systems can be observed at 40–60 atmospheres, where there are small reductions in the sedative properties of propofol (Tonner et al., 1992) and changes in the affinity of hemoglobin for oxygen (Reeves and Morin, 1986). The major effect of pressure changes within the depths usually encountered by man is therefore on gas properties. Several simple gas laws were derived from the ideal gas law (PV = nRT) and are currently used under conditions associated with diving (i.e., Boyle’s law, Dalton’s law, and Henry’s law). A column of liquid exerts a pressure that is proportional to its height, density, and the acceleration of gravity. At sea level the ambient pressure is 1 atmosphere absolute (ATA). For each 10 m depth in water a diver is exposed to an additional pressure of 1 ATA Because gases are compressible the lung may therefore experience significant changes in volume, especially during hasty ascents (Pendergast et al., 2015).

### Temperature

*Thermal conductivity* is the property of a material to transfer heat from one molecule to another through a liquid, solid, or gas. Typically, heat loss in the human body is mainly related to radiative and convective factors, so temperatures within 18–24°C are comfortable for a body surrounded by air. Conversely when in water, a short period of time exposed to that temperature range can cause great discomfort or worse. Thermoneutrality in complete submersion, defined as the temperature at which there is no heat transfer between the water and the body, is 28–30°C. When the water is colder, suitable protection is needed to reduce heat loss. Similarly, active cooling may be required for diving in warmer waters. For respiratory heat loss, the important parameter is thermal or heat capacity. *Heat capacity* is higher for diatomic molecules like N_2_ and O_2_ than for monoatomic gases such as helium or argon. Because of the high thermal capacity of gases under pressure, breathing mixtures during deep diving in cold water are sometimes preheated (Brubakk et al., 2003).

### Optical Phenomena

When underwater, it is extremely difficult to focus images on the retina due to the similar refractive index between the cornea and water. Therefore, an air interface by means of a mask or special glasses is required, creating two interfaces with different refractive powers (water vs. glass and glass vs. air). Kent (1966) found, however, due to this layering of interfaces, that except at short ranges, both size and distance were overestimated underwater compared to normal viewing under above water conditions. Since the refractive index of the water is four-thirds that of air, the optical (apparent) distance of a target under water is three-fourths of that in air (Ono et al., 1970). Immersed objects appear about 30% larger and closer, due to the distorted perception.

This distortion derives from the passage of the rays of light from the water into the gas environment within the mask, due to the refraction at the interface where the light speed increases, as described by Snell’s law:

$$\begin{array}{c} \left(\frac{Sin\theta_{1}}{Sin\theta_{2}}=\frac{v_{1}}{v_{2}}=\frac{\lambda_{1}}{\lambda_{2}}\right), \end{array}$$

*𝜃* as the angle measured from the normal of the boundary, *v* as the velocity of light in the respective medium (SI units are meters per second, or m/s), λ as the wavelength of light in the respective medium.

Moreover, refraction alters the form and location of the image resulting in variations in the perceived lateral position as the angle of incidence of the light rays increases. These physiological alterations may disturb the divers’ hand-eye coordination and motor skills. However, individuals own notable abilities to adjust to changes in different conditions, such as light, color and optical distortions (Luria and Kinney, 1970). Light traveling in the air does not considerably modify its spectral composition, but light transmission through water can limit color recognition (Kinney et al., 1967). Furthermore, both the quantity and quality of the change depend on the particular body of water involved. Pure water has its greatest transmittance at 480 mμ in the blue-green region of the spectrum. Kinney et al. (1967) determined empirically the underwater visibility of colored paints, both fluorescent and non-fluorescent, in different waters. The easiest color to see when the water clarity increases ranges from yellow-green to green to blue-green. Conversely, gray and black represent the two colors hardest to see in a water environment with a natural illumination. Moreover, other colors whose spectral components are absorbed by the water are orange and red in clear water and blue and green in murky water, implying poor visibility (Kinney et al., 1967).

## Man and Water

We can summarize the human body immersion in the water in three different aspects:

(a) partial immersion of the human body, such as face, lower limbs, body without head; (b) full immersion (submersion) breath-hold diving at various lung volumes; (c) submersion with auxiliary breathing apparatus to provide an appropriate gas mixture (oxygen, air, helium-oxygen, helium-oxygen-nitrogen, oxygen-hydrogen etc.) (Pendergast et al., 2015).

### Partial Body Diving

Immersion results in a substantial modification on the dynamics of venous return toward the intrathoracic vessels and the right part of the heart. The increased intrathoracic blood flow is due to the reduction of blood flow in the peripheral veins. This phenomenon is induced by the increase in surrounding pressure of the submerged limb and, assuming average ocean surface water temperature 17°C, the active vasoconstriction of veins triggered by the loss of heat due to the high thermal conductivity of water. Swimming with head above water induces an estimated 500 ml increase in intrathoracic blood volume. Blood-shift due to immersion is responsible for the expansion of the right heart cavities and the neuroendocrine modifications shown after immersion. Specifically, immersion activates stretch receptors located in the atrial wall, stimulating the secretion of atrial natriuretic peptide (ANP). This explains the strong natriuresis and diuresis that occurs during immersion (Kollias et al., 1976; Lundgren and Miller, 1999).

Facial immersion in cold water results in the *diving reflex*, consisting of: apnoea, bradycardia, vasoconstriction, increased mean arterial pressure and splenic contraction (Schagatay et al., 2001, 2005; Lemaitre et al., 2015). The diving response in humans can be influenced by several factors, and water temperature is one of the most important. Gooden (Gooden, 1994) summarized a series of findings focusing on face immersion with breath-holding and showed a well-defined inverse relationship between the water temperature and the degree of the diving-induced bradycardia. Recently Lemaitre et al. (2015) elucidated the many similarities and the functional purposes shared by trigeminocardiac reflex (TCR) and diving reflex (DR). Decreasing water temperature proportionally increases the response of the TCR; this is a factor in the intensity of stimulation of the DR (Lemaitre et al., 2015).

### Breath-Hold Diving

Immersion with voluntary breath holding requires no equipment but induces extreme changes in cardiovascular physiology. In breath-hold diving, two main factors affect the physiological changes of the human body: the *time* of breath suspension and the *depth* of submersion (Lundgren and Miller, 1999). Individuals have reached depths greater than 200 m using weight-assisted descent and ascent facilitated by balloon. Basing on the concept of the thoracic squeeze these dives reach greater depth than predicted by Boyle-Mariotte’s law. Until a few years ago, it was hypothesized that the maximum depth reached after a forced inspiration was determined by the ratio between the total lung capacity (TLC) and residual volume (RV). During progressively deeper submersion lung volume is compressed from TLC toward RV and it was hypothesized that overstepping this predicted depth, the hydrostatic pressure should induce pulmonary vascular rupture. Based on this concept, divers could not have reached more than 30–35 m depths, while in fact in 1903 a Greek sponge fisherman, Georgios Stattis, descended to 71 m holding his breath to recover the anchor of an Italian ship, the “Regina Margherita” (Bosco et al., 2007). However, hemoptysis can occur during breath-hold diving, and fatal pulmonary hemorrhage has been described (Mijacika and Dujic, 2016). Furthermore, rupture of the pulmonary capillaries can allow air to enter the vasculature and cause cerebral arterial gas embolism (Kohshi et al., 2000, 2005).

The possibility of reaching greater depths is due to a physiological compensation of physical laws; blood from peripheral circulation is shifted to the chest and being an incompressible fluid, it modulates the expected reduction of the pulmonary volume below the residual volume, and can further compress alveolar gases. In this way, a simpler application of physical laws in breath-hold deep diving does not apply. The intrathoracic blood volume increase, investigated with different methods (plethysmography, bioelectrical impedance analysis, scintigraphy, radiography), and is nowadays hypothesized to exceed 2 l (Arborelius et al., 1972; Robertson et al., 1978; Data et al., 1979; Lanphier, 1987). Additionally, the classic diving reflex, induced by face immersion leads to bradycardia. This physiological response appears in many different species both mammals and birds, although varying in terms of time and intensity. Breath-holding at rest, out of water, induces non-significant changes in heart rate; as well as immersion itself do not cause bradycardia while swimming and snorkeling (breathing through an aerator tube). Breath-hold swimming, even on the surface, instead causes pronounced bradycardia (Ferrigno et al., 1986; Andersson et al., 2002; Ferretti and Costa, 2003; Lindholm and Lundgren, 2008). Severe hypertension has also been observed in championship breath hold divers during dives in a hyperbaric chamber (Ferrigno et al., 1997).

### Submersion with Self-Contained Breathing Apparatus

Diving with self-contained breathing apparatus induces physiological changes in the subject, with possible pathological events. Major adjustments are those concerning respiratory mechanics (Camporesi and Bosco, 2003). Simple body immersion in water results in a significant reduction of vital capacity, residual volume, lung distensibility, and functional residual capacity due to the increased venous return and resulting pulmonary blood content. These changes reduce respiratory performance and increase tidal volume, while maintaining the respiratory minute volume constant (Moon et al., 2009).

In addition, the pressure of the air provided by the breathing apparatus is influenced by the position of the diver’s body while diving. In particular, when the diving regulator is positioned higher in the water column than the lung, it delivers air at a lower pressure and thus increasing the inspiratory work. This phenomenon is not specific only for scuba diving, but it also exists in partial body immersion in a vertical position.

When breathing gas with a fixed oxygen concentration, alveolar and arterial oxygen partial pressures increase with depth. This tends to attenuate ventilatory chemosensitivity (Dunworth et al., 2017) and facilitate CO_2_ retention. Seasoned divers indeed become less sensitive to high CO_2_ partial pressures.

The nitrogen distribution between the various compartments of the organism are characterized by latency that at the end of the submersion leads to a nitrogen excess in the body with respect to pre-diving levels. Tissue supersaturation with inert gas such as nitrogen or helium during decompression is the mechanism for decompression sickness (DCS) (Vann et al., 2011). If the rate of ascent is too fast in relation to the quantities of nitrogen absorbed then the solubilized nitrogen will be released into gaseous phase with bubble formation in the circulating blood and tissues (Bosco et al., 2010; Arieli and Marmur, 2013). For this reason, decompression procedures have been developed, in which decompression is staged, in order to allow inert gas to be eliminated via the lung at a rate consistent with a minimal risk of bubble formation.

## Diving Disorders

Subaquatic environments expose divers to a variety of pathologies, varying from *drowning*, probably the most frequent complication induced by inhalation of water and resulting in asphyxia, to *transient syncope*, i.e., loss of consciousness usually resulting from systemic or district hypoxia during breath-hold diving. Sometimes it is due to or associated with hypercapnia. There might be multiple causes for drowning and not all causes are related to the submerged environment: the most notable causes are immersion barotrauma and immersion pulmonary edema (IPE). More complex and unique pathologies derive from *decompression*. Decompression disorders are defined as pathological events that result from a reduced environmental pressure caused by the development or penetration of gaseous bubbles within the blood and tissues.

### Barotrauma

Barotrauma is a common disorder among professional divers. It is caused by volume changes (increase or reduction) of gas placed in spaces within, or adjacent to, the body. This results in tissue damage (Strauss, 1979). The aforementioned Boyle’s Law describes the changes of gas volume in relation to the pressure applied to it. The greater the pressure applied, the bigger the gas compression. Conversely, as the pressure decreases the gas volume is allowed to expand. Hence, the gas compression/expansion derived from this mechanism, widely enhances the possibility of damage to tissues (Brubakk et al., 2003).

While the diver is submerging, the pressure applied to his body increases; the diminished volume of the surrounded gases will result in barotrauma of descent. Differently, the related drop in pressure applied to the emerging diver, will result in barotrauma of ascent due to gas expansion. Though descent and ascent barotrauma affect the same sites, the clinical symptoms are different, considering the different pathophysiological changes. Gas within the body can be enclosed in distensible or in bony tissues. However, the possibility that barotrauma occurs when gases are surrounded by solid organs (i.e., sinus and middle ear) is likely. Similarly, when the gas is trapped in artificial sites (i.e., face mask) the changes of pressure equally result in potential barotrauma (Strauss, 1979).

Scientific literature divides barotrauma considering the affected organs. *Ear barotrauma* is the most common type of barotrauma and can cause a range of issues from mild hyperemia of the tympanic membrane to its actual rupture (Sadri and Cooper, 2017). It is due to the discrepancy in pressure between the outside environment and the middle ear. Insufficient pressure equalization can result in ear pain during descent, and subsequent Valsalva maneuvers may cause acute, spinning vertigo, disorientation, nausea, and vomiting (Brubakk et al., 2003). Similarly, nasal functionality and equalization maneuvers are primary to avoid *sinus barotrauma*. Rosińska et al. (2015) identified inflammation of the mucous membrane, nasal or sinus polyps, as well as nasal septum curvature and nasal concha hypertrophy as risk factors for sinus barotrauma. Indeed, since the gas is blocked in the sinus cavities the increase of pressure brings to absorption of the air hosted in the sinus with void development (Strauss, 1979). During descent or ascent, obstruction of the sinus ostia or eustachian tube generates a pressure differential that can result in pathological manifestation such as hemorrhage (Strauss, 1979), headache and paresthesia in the area innervated by the infraorbital nerve (Rosińska et al., 2015).

*Pulmonary barotrauma* is the most severe of the barotraumas and causes the most concern in all types of diving operations (Strauss, 1979). It represents one of the commonest cause of arterial gas embolism (AGE). It may be considered as barotrauma of descent and barotrauma of ascent. As the depth increases the total lung volume decreases up to approach the residual lung volume. Since the lung compressibility reaches its limit deeper submersion will cause lung collapse, preceded by pulmonary congestion, edema and hemorrhage. Although rare, pulmonary barotrauma during decompression (Wolf et al., 1990) may occur due to breath holding or focal gas trapping due to airway obstruction. Significant air trapping and history of spontaneous pneumothorax are also causes for concern during hyperbaric oxygen therapy and mandate a careful analysis of potential benefit from hyperbaric medicine oxygen therapy vs. the associated risk (UHMS, 2014).

*Mask barotrauma*, also known as “mask squeeze” or facial barotrauma, is caused when the diver inadvertently closes his nasopharynx, causing a pressure differential between the gas trapped within the mask worn by the diver and his face. Hence, when the diver descends in the water, periorbital soft tissues are forced into the mask, causing edema and bleeding (Lynch and Deaton, 2014). A case report defined the subconjunctival hemorrhage among the symptoms of the ocular barotrauma of descent (Ergözen, 2017). It is possible to prevent this type of barotrauma wearing mask rather than goggles, also during free-diving, and to apply correct techniques of mask equalization. *Gastrointestinal barotrauma* is caused by the augmented volume of the gas stored in the gastrointestinal tract. Gas expansion during the ascent phase can result in symptoms such as cramping, pain, distention, and bloating. A therapeutic approach may modulate food and liquid ingestion before diving (Lynch and Deaton, 2014). *Dental barotrauma* happens when artificial gas-filled spaces (i.e. caps, crowns, root canals) are damaged due to gas expansion during ascent. As reported by Zadik and Drucker (2011) dental barotrauma occurs while the diver is ascending. At the surface, after completing the dive, dental barotrauma may result in tooth fracture, restoration fracture, and reduced retention of dental restoration (Zadik, 2009).

### Immersion Pulmonary Edema (IPE)

This condition, also referred to as swimming induced pulmonary edema (SIPE), was first described in healthy swimmers and divers by Wilmshurst et al. (1989). SIPE presents during a swim or dive with acute onset of dyspnea and cough, often productive of bloody sputum. Other manifestations include hypoxemia and bilateral pulmonary infiltrates. Many patients with SIPE require hospitalization. In Wilmshurst’s series these individuals, many of whom subsequently developed hypertension, had an exaggerated hemodynamic and vasoconstrictive response to cold applied to the skin (packing of head and neck in towels soaked in ice-cold water), and it was hypothesized that during immersion in cold water the increase in afterload precipitated heart failure. This syndrome was subsequently reported in naval recruits (Weiler-Ravell et al., 1995; Shupak et al., 2000; Mahon et al., 2002; Lund et al., 2003), triathletes (Miller et al., 2010) and many non-divers and non-elite swimmers (Peacher et al., 2015).

Uncertainty existed as to how the mechanism initially proposed could apply to exceptionally fit individuals such as triathletes and special naval forces trainees. Water aspiration was implausible in most cases, and increased permeability as in acute respiratory distress syndrome (ARDS) did not seem likely. Recent invasive hemodynamic investigations have been performed in volunteers with and without a SIPE history during submersed exercise in cold (20°C) water. SIPE-susceptible individuals have higher pulmonary artery and pulmonary artery wedge pressures than controls (Moon et al., 2016b), which solidified the notion of a hemodynamic mechanism for the capillary leak. However, the reason for the higher vascular pressures is still unknown. Medical predisposing factors in some individuals include hypertension, cardiomyopathy left ventricular hypertrophy (LVH) and valve disease (Peacher et al., 2015). It is now hypothesized that a common mechanism for SIPE is the combination of heavy exercise, mild to moderately stiff left ventricle (mildly abnormal LV diastolic properties) and central blood redistribution due to immersion/submersion, causing excessively high filling pressures.

Deaths have been reported due to SIPE (Cochard et al., 2005). Indeed, a significant portion of deaths during triathlon events occur in individuals with LVH, suggesting that fatalities are often precipitated by SIPE (Moon et al., 2016a).

Preventive measures include avoidance of fluid loading before a competitive swimming event, which presumably attenuates the central blood redistribution. Sildenafil administered before a swim in cold water lowers pulmonary vascular pressures (Moon et al., 2016b) and a case report supports the notion that sildenafil administered before a triathlon swim may prevent SIPE in predisposed individuals (Martina et al., 2017). It should be noted that sildenafil may not be safe in divers who breathe oxygen tensions greater than 1 ATA, as the drug causes cerebral vasodilatation and is likely to predispose to convulsions caused by CNS oxygen toxicity (Demchenko et al., 2009). Data from Blatteau et al. (2013) also reported that sildenafil increase the risk of DCS.

### Decompression Illness

Bubble formation in blood stream or tissue during or after a decrease in environmental pressure (decompression) is defined decompression illness (Vann et al., 2011). There are two fundamental decompression pathologies in the underwater environment, characterized by different pathophysiological genesis: *decompression sickness* (DCS) and *arterial gas embolism* (AGE). Each pathophysiology involves different mechanisms that can result in damages in the particular tissue affected via: direct blockage of blood flow; tissue damage or compression by direct mechanical effect; endothelial damage resulting in impaired regulation of the endothelium-mediated microcirculation and capillary leak; secondary effects mediated by immune or inflammatory pathways.

In decompression sickness (DCS) bubbles form, due to insufficient time to achieve decompression and gas elimination from the lungs. Bubbles form in tissues or in the blood-stream when local inert gas pressure exceeds a threshold value and inert gas comes out of solution. The exact biophysical mechanism is not clear, but there is agreement that in the diving environment supersaturation occurs when the rate of ambient pressure reduction exceeds the rate of inert (usually nitrogen) gas washout from tissues. The different manners of diving (i.e., breath-hold or scuba) may also induce different physiological or pathophysiological effects, depending on the altitude at which the immersion is performed: sea level immersion, or at high altitude, with or without previous changes induced by acclimation (Egi and Brubakk, 1995; Bosco et al., 2001; Clarke et al., 2015).

Traditional classification of DCS comprises type 1, including pain and cutaneous manifestations, plus subjective symptomatology; and type 2, where tingling, paresthesia and numbness will appear, at times with muscle weakness and mental and motor abnormalities.

**FIGURE 1:**
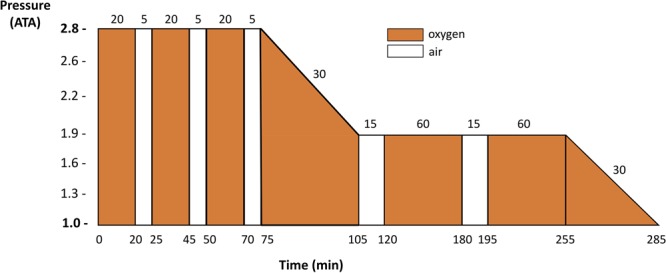
US Navy Table 6 profile commonly used in clinical routine for DCS recompression procedure for recompression of divers with DCS or arterial gas embolism. Redrawn from Navy Department (2016).

Treatment begins at sea level with 100% oxygen breathing and recompression therapy as soon as possible. While around the world various recompression procedures are implemented, since their introduction in the 1960’s the US Navy oxygen treatment tables with initial recompression to 18 m (2.8 atmospheres absolute) and administration of 100% O_2_ (US Navy Table 6, **Figure 1**) have been the most widely used. They are highly effective if administered without excessive delay after development of symptoms (Vann et al., 2011). Other procedures used in Europe and elsewhere follow the same general principles of pressure and oxygen breathing.

*Arterial gas embolism* might cause a more overt and extensive symptomatology but the treatment will converge to recompression and oxygen breathing. Embolism results from rupture of alveoli during ascent from a dive resulting in entry of air into the bloodstream. The most common cause of embolism in underwater activities is a rapid ascent with breath holding or regional gas trapping in the lung due to lung pathology. Therapeutic remedies are similar both for DCS and air embolism, both utilizing O_2_ breathing and recompression. It is recommended to use only Table 6.

Recently, certain interventions such as exercise are viewed as protective form of preconditioning reducing the venous gas emboli quantity (Dujic et al., 2008). Moreover, oxygen pre-breathing has shown protective effect on bubble production and platelets activation; deeply, it has been shown correlation among oxygen partial pressure breathing and modulation of reactive oxygen species and related antioxidant enzyme (Landolfi et al., 2006; Bosco et al., 2010; Morabito et al., 2011).

Thom et al. (2015) offered a novel opportunity to explore associations of circulating microparticles and neutrophil activation that may contribute to development of DCS. Further work will be needed to explain and clarify DCS pathophysiology.

### Nitrogen Narcosis

Nitrogen does not participate in any metabolic process and therefore is defined as an inert gas; hence, nitrogen levels in the body are determined solely by the atmospheric pressure (Freiberger et al., 2016). During air breathing at sea level, the body is therefore “saturated” with nitrogen. During immersion with underwater air breathing, nitrogen follows its alveolar-arterial pressure gradient passing from the alveoli to the lung capillaries, and hence to peripheral tissues. Reversal occurs in the ascending phase and after the dive.

Narcosis while diving (also known as nitrogen narcosis, inert gas narcosis, raptures of the deep, Martini effect) is a pathology that arises following a considerable increase in nitrogen partial pressure and consists of alteration in consciousness and neuro-sensory state. Manifestations consist of distorted perceptions, hallucinations, difficulty in concentrating and performing elementary tasks, confusion and loss of consciousness. There is a wide inter-individual susceptibility to the effects of hyperbaric nitrogen, which can be augmented by hypercapnia (Freiberger et al., 2016). Reducing the nitrogen partial pressure (basically reducing the depth) causes rapid recession of the symptoms, but recent experimental data on a controlled group of healthy divers (Balestra et al., 2012) supports the view that nitrogen narcosis can interact with the production and release of a variety of neurotransmitters and might require substantial time delay to recover after exposure. In this last study, the authors utilized Critical Flicker Fusion Frequency (CFFF) measurements to time the onset and resolution of N_2_ narcosis after a dive. The impairment of CFFF persisted 30 min after surfacing and the narcotic effect dissipated only after this time delay.

### Oxygen Toxicity

The use of enriched air or closed-circuit rebreather during diving, can potentially result in oxidative injury affecting the brain, lungs and eyes. However, lung and brain injuries develop mainly because of the toxic effects of oxygen free radicals on lung parenchyma, airway and brain (Demchenko et al., 2007, 2008; Buzzacott and Denoble, 2017). Central nervous system oxygen toxicity (CNS-OT) is one of the most harmful effects of Enriched Air Nitrox (EAN) diving and it is related to oxidative stress and inflammation (Fock et al., 2013; Arieli et al., 2014). CNS-OT may cause convulsions similar to epileptic seizures, with sudden loss of consciousness, and other symptoms such as nausea, vomiting, palpitations, visual field constriction, tinnitus and auditory hallucinations. Recent studies have shown that seizures due to CNS O_2_ toxicity are probably preventable by conventional anticonvulsant drugs (Demchenko et al., 2017).

A breathing mixture with sufficiently high PO_2_ can also induce pulmonary oxygen toxicity (Arieli, 2006; Balestra and Germonpre, 2006). Indeed, exposure to HBO_2_ may lead to temporary reductions in pulmonary function (Van Ooij et al., 2013). While pulmonary mechanics are altered during hyperbaric exposure due to gas density effects (Hrnčíř, 1996), there are also data on possible long-term effects of diving on respiratory mechanics. A spirometric investigation revealed a long-lasting impairment of the conducting function of the small airways in humans accustomed to perform deep dives. Interestingly, these results were observed in subjects using oxygen but also in subjects using air as breathing gas (Tetzlaff et al., 1998). In addition, a small but significant reduction in FVC was observed 24 h after a single dive to a depth of 50 m while breathing air (Tetzlaff et al., 2001), suggesting that an increment in airway resistance occurred and persisted long after the dive.

Recent experimental work has been performed on animals to investigate the possible effects of hyperoxic hyperbaric exposure on the mechanics of respiratory parameters. Previously, it has been demonstrated that the exposure to hyperbaric hyperoxia increased respiratory system elastance and both the “ohmic” and viscoelastic components of inspiratory resistances, probably due to increased nitrogen reactive species production and iNOS activity (Rubini et al., 2013, 2014). Conversely, a single exposure to hyperbaric air at 2.5 atmospheres for 90 minutes does not affect either the elastic or the resistive respiratory system properties (Rubini et al., 2014). More researches using new experimental technologies are requested to continue the predictive studies on work capability and physiological effects under pressure (Lambertsen, 1976).

### High Pressure Nervous Syndrome

High Pressure Nervous Syndrome (HPNS) is a set of motor and sensory symptoms that appear during deep helium diving, typically at depths deeper than 150–180 m. Subjects affected by HPNS complain of general illness, intentional and postural tremor, asthenia, drowsiness (or otherwise insomnia), nightmares, fasciculation or muscle twitches and myoclonic contractions (Bennett et al., 1981). Electroencephalographic anomalies are also observed, which include a generalized increase in slow activity and a decrease in frequency at higher frequencies. This pathology appears to be produced by the direct effect of high environmental pressure on cells, through modifications in neurotransmitter function (Rostain and Balon, 2006).

## Conclusion

Underwater physiology research today, considering its tradition, focuses on the prevention of underwater accidents, with pharmacological and physical approach as preconditioning (Bosco et al., 2010; Morabito et al., 2011) to reduce the bubble formation, inflammation and the risk of DCS (Castagna et al., 2009; Germonpré and Balestra, 2017). Long sojourning and efficient maneuverability and working capabilities can only be achieved with underwater habitats capable of being maintained for weeks and months with human safety factors, appropriate rotation of selected crews and safe decompression protocols. It is conceivable that fast evacuation from such environments might require rapid “at depth” extraction of the crew with capability of surface decompression in appropriate support vessels. Such specialized and costly stations at depth could only be justified for surveillance of mineral extraction resources, oil pumping stations or military purposes at significant depths, where breathing gases might be tailored to the specialized needs.

## Notes

**Archimedes’ law**: An object partially or wholly immersed in a fluid is buoyed up by a force equal to the weight of the fluid displaced by the object.
**Boyle’s law**: the absolute pressure exerted by a given mass of an ideal gas is inversely proportional to the volume it occupies if the temperature and amount of gas remain unchanged within a closed system.
**Dalton’s law**: in a mixture of non-reacting gases, the total pressure exerted is equal to the sum of the partial pressures of the individual gases.
**Henry’s law**: the amount of dissolved gas is proportional to its partial pressure in the gas phase.

## Author Contributions

GB substantial contributions to the design of the work and drafting the manuscript. AR reviewed the literature and wrote the manuscript. RM wrote the paper and revised it critically for important intellectual content. EC contributed to review and write the manuscript, editing of the language. All authors critical reviewed the final draft of the manuscript and approved the version submitted.

## Conflict of Interest Statement

The authors declare that the research was conducted in the absence of any commercial or financial relationships that could be construed as a potential conflict of interest.
